# Expression of Concern: Hsa_circ_0000673 is down-regulated in gastric cancer and inhibits the proliferation and invasion of tumor cells by targeting miR-532-5p

**DOI:** 10.1042/BSR-20180538_EOC

**Published:** 2021-02-16

**Keywords:** cell proliferation, gastric cancer, Hsa_circ_0000673, Invasion, miR-532-5p

The Editorial Office has been made aware of potential issues surrounding the scientific validity of this paper, hence has issued an expression of concern to notify readers whilst the Editorial Office investigates.

